# Lanreotide Induces Cytokine Modulation in Intestinal Neuroendocrine Tumors and Overcomes Resistance to Everolimus

**DOI:** 10.3389/fonc.2020.01047

**Published:** 2020-07-07

**Authors:** Concetta Sciammarella, Amalia Luce, Ferdinando Riccardi, Carmela Mocerino, Roberta Modica, Massimiliano Berretta, Gabriella Misso, Alessia Maria Cossu, Annamaria Colao, Giovanni Vitale, Alois Necas, Jan Fedacko, Marilena Galdiero, Pierpaolo Correale, Antongiulio Faggiano, Michele Caraglia, Anna Capasso, Anna Grimaldi

**Affiliations:** ^1^Department of Precision Medicine, University of Campania “Luigi Vanvitelli”, Naples, Italy; ^2^Department of Clinical Medicine and Surgery, University “Federico II” of Naples, Naples, Italy; ^3^Oncology Unit, AORN Cardarelli, Naples, Italy; ^4^Department of Medical Oncology, Centro di Riferimento Oncologico, Istituto Nazionale Tumori CRO, Aviano, Italy; ^5^Laboratory of Precision and Molecular Oncology, Institute of Genetic Research, Biogem Scarl, Avellino, Italy; ^6^Laboratory of Geriatric and Oncologic Neuroendocrinology Research, Istituto Auxologico Italiano, IRCCS, Milan, Italy; ^7^Department of Clinical Sciences and Community Health (DISCCO), University of Milan, Milan, Italy; ^8^CEITEC – Central European Institute of Technology, University of Veterinary and Pharmaceutical Sciences Brno, Brno, Czechia; ^9^1st Department of Internal Medicine, Centre of Excellency for Atherosclerosis Research, University of Pavol Jozef Safarik, Košice, Slovakia; ^10^Department of Experimental Medicine, University of Campania “Luigi Vanvitelli”, Naples, Italy; ^11^Medical Oncology Unit, “Bianchi-Melacrino-Morelli” Grand Metropolitan Hospital, Reggio Calabria, Italy; ^12^Department of Experimental Medicine, Division of Endocrinology, Sapienza University of Rome, Rome, Italy; ^13^Department of Oncology, Livestrong Cancer Institutes, Dell Medical School, The University of Texas, Austin, TX, United States

**Keywords:** neuroendocrine tumors, cytokines, somatostatin analogs, mTOR–mammalian target of rapamycin, drug-resistance

## Abstract

Somatostatin analogs mantain their major role in the treatment of patients with advanced neuroendocrine tumors (NETs) and have multiple modulatory effects on the immune system. Here, we evaluated the effects of lanreotide treatment on expression of Th1, Th2 cytokine patterns in serum of patients with NETs and in bronchial and pancreatic NET cell lines. Our results showed that lanreotide treatment promoted a Th1 cytotoxic immune-phenotype in patients with NETs originated by intestinal sites. Similar results were obtained also *in vitro* where lanreotide induced expression of Th1 cytokines only in pancreatic and not in bronchial-derived NET cell lines. It seems, therefore, that cytokinomics can represent a useful tool for the identification of tumor biomarkers for the early diagnosis and evaluation of the response to therapy in NET patients. To avoid the drug-resistance induced by everolimus (mTOR inhibitor), we made the pancreatic NET cell line resistant to this drug. After treatment with lanreotide we found that the drug reduced its viability compared to that of sensitive cells. These data may have direct implications in design of future translation combination trial on NET patients.

## Introduction

Neuroendocrine Neoplasms (NENs) are heterogeneous, with increasing incidence in the last decades arising from altered stem cells programmed to evolve in ultimate lineages scattered with secretory granules and the ability to produce hormones (neuropeptidic, neurotransmitter and neuromodulator with endocrine, autocrine and paracrine action) that lead to carcinoid syndrome. Our study was performed on neuroendocrine tumors (NETs) that are differentiated NENs. Although the incidence of NETs is largely underestimated and comprises <2% of gastrointestinal malignancies, their prevalence appears to be greater than that of stomach as well as pancreatic adenocarcinomas (1, 2). Most common NETs occur in the gastro-entero-pancreatic (70%) and respiratory (25%) systems even though they may rise from any tissue and body district, including genitourinary tract, skin (merkelioma), thyroid, adrenal, nervous ganglia etc. (1). Most patients with NETs are diagnosed with advanced diseases and the mortality rate is 50% within five years (3, 4). Their treatment is based upon surgical resection for localized tumors or for NETs with a regional diffusion and, to alleviate the symptoms, in metastatic or high-grade tumors (5, 6). Unfortunately, the symptoms associated with this tumor may be nonspecific or absent. In fact, the diagnosis of NETs is often delayed and becomes necessary to use medical therapy. Being slowly growing tumors, patients are often subjected to long-lasting treatments (6). In the last twenty years, the best therapeutic approaches to these tumors have been based on the use of somatostatin analogs (SSAs) (octreotide, lanreotide) and, later on, mammalian target of rapamycin (mTOR) inhibitors such as everolimus (7, 8) have also shown their efficacy in the treatment of patients with these malignacies. Somatostatin receptor (SSR) subtypes are 5 (SSR 1–5) (9, 10) and somatostatin, binding to its receptors, activate both antisecretory and antiproliferative effects. The antisecretory effects are mainly due to inhibition of exocytosis mainly induced by the decrease of intracellular cAMP and calcium levels. Antiproliferative effects are induced by cell cycle arrest or apoptosis activated by protein tyrosine phosphatases or through the inhibition of the secretion of growth factors (11, 12). The receptors can also form dimers thus having complex effects on the cells through the activation of alternative signal transduction pathways (13). Most of these NETs express SSR, predominantly subtypes 1, 2, and 3 with an inverse correlation with the grade of differentiation of the tumor (14).

Octreotide and lanreotide are the first-generation SSAs and show a high binding affinity to SSR2 and 5, while pasireotide, which is a second-generation SSA, has high affinity for multiple SSRs (SSR5 > SSR2 > SSR3 > SSR1) (15). Lanreotide, in details, is currently approved for treatment of NETs and has a relevant cytostatic and antisecretive effect. Two important phase III trials, PROMID (placebo-controlled, prospective, randomized study in patients with metastatic neuroendocrine midgut tumors) and the CLARINET (controlled study of lanreotide antiproliferative response in neuroendocrine tumors), have been performed on patients with midgut and gastroenteropancreatic NETs, respectively. In the PROMID trial, 85 patients with NET were randomized to receive either octreotide or placebo. Octreotide was associated to a significant longer time to tumor progression compared to the placebo (14.3 months within the octreotide group and 6.0 months in the placebo group) and lower tumor progression rates (16). CLARINET assessed the SSA lanreotide in patients with advanced, G1/G2 differentiated, nonfunctioning, somatostatin receptor-positive NETs and documented disease progression status. In that study, lanreotide was linked to significantly prolonged progression-free survival (PFS) compared to the placebo (estimated rates of PFS at 24 months 65.1% in the lanreotide group and 33.0% in the placebo group) (17). Long-term results from both trials demonstrated the long-lasting control of the disease for octreotide and poor side effects in prolonged treatment for lanreotide (18). On the other hand, mTOR is an intracellular effector involved in cell survival, proliferation and metabolism regulation (19) acting through Thr^389^phosphorylation of translational regulator, ribosomal protein S6 kinase β-1 (S6K1) and phosphorylation of eukaryotic translation initiation factor 4E-binding protein 1/2 (4eBP1/2), which, respectively, induce mRNA biogenesis and cap-dependent translation, increasing protein synthesis, cell growth and proliferation (19, 20). mTOR expression was observed significantly higher both in primary lesions and in metastases from NETs. This finding is coherent with a driver role of mTOR pathway activation in NET tumorigenesis (21). It is evident that the inhibition of this signaling pathway represents an excellent pharmacological target. Currently, based on the results of several clinical trials, everolimus (a pharmacologically active inhibitors of mTOR) is approved for the treatment of advanced pancreatic, gastrointestinal and lung NETs (22, 23). Moreover, everolimus should be considered a valid therapeutic option for extrapancreatic NETs (24). However, one-third of NET patients show primary insensitivity (primary resistance) to treatment with everolimus, while in others the disease is initially stabilized and then develops resistance (acquired resistance) and disease progression; this could depend on the genetic instability and the heterogeneity of tumor cells (25). Lastly, some indications suggest that combination of mTOR inhibitors with other target-based drugs, including dopamine agonists and SSAs could be a promising strategy in NET treatment (26, 27). Recent studies have highlighted the cell interactions between the tumor and the immune system in the tumor microenvironment; these interactions allow the malignant cells to use the local mechanisms present in the latter, preventing the activation of the functions of the immunological effectors and, thus, protecting the tumor from the attack of the immunological effectors (28). Two mechanisms of immunosuppression have been highlighted: (i) alteration of the genes (oncogenes) of the tumor cells and (ii) adaptive immuno-resistance supported by tumor-specific T cells (28). Several studies performed on somatostatin and its analogs have also shown that tumor cells synthesize cytokines that favor escape from immunosurveillance and may also act as tumor growth factors (29–33).

Somatostatin is a very pleiotropic molecule able to exert different effects on a number of immune cells where different SSRs are expressed. Firstly, somatostatin by itself is able to stimulate the production of Interleukin-1β (IL-1β) and Tumor Necrosis Factor α (TNFα) (34), two dominant pro-inflammatory cytokines which are critically involved in the activation of both inflammation as well as immune reactions consequent to specific tissue damages. In this light, one of its analogs, octreotide, whose binding activity is limited to SSR2 and 5, seems to be able to inhibit the production of TNFα (31) and to increase the production of Interleukin-10 (IL-10) in patients with autoimmune diseases (32). In another study, octreotide and pasireotide also showed the ability to decrease the production of both Interferon γ (INF-γ) and Interleukin-2 (IL-2) by *in vitro* stimulated T cells (33). All together, these results suggest that the two SSR2 stimulating agents seem to promote the induction of a type 2 helper immunophenotype (Th2) that drives the immune reaction from cell mediated (Th1) toward a humoral response. In this way, it can be hypothesized that SSR agonists may interfere with both tumor microenvironment and immune reaction. On these bases, we believe that cytokinomics can represent a useful tool to study either inflammatory and/or immunological issues in patients with advanced NET under treatment with lanreotide aimed to detect potential biomarkers of response and new therapeutic targets for these patients. Moreover, we have evaluated the effects of lanreotide on Th1 and Th2 functional profile on NET cell lines (typical bronchial NET NCI-H727 and pancreatic NET BON-1) and in patients with advanced NETs by evaluating specific cytokine patterns (IL-2, IL-4, IL-6, IL-10, IFN-γ, and TNFα). By taking in consideration that PI3K/AKT/mTOR inhibitors, like everolimus, are known immunesuppressive drugs used in the prevention of bone marrow transplantation and are currently used in the treatment of not resectable pancreatic NET and bronchial carcinoids, we have also evaluated whether treatment with lanreotide may also be used to revert resistance to everolimus in NET cell lines.

## Materials and Methods

### Cell Cultures

BON-1 cells were a kind gift from University of Turin, San Luigi Hospital, Orbassano. BON-1 cell line is the most widely used *in vitro* GEP-NET cell line model. In fact, this is an easy-to-handle immortalized cell line that allows a high rate of experimental reproducibility. NCI-H727 cells were provided by American Type Culture Collection (ATCC). BON-1 R (everolimus-Resistant) cells were obtained after chronic treatment with everolimus for eight weeks. During treatment, increasing drug concentrations (from 1.25 to 10 μM) were added to the culture medium every 48 h, doubling its concentration every two weeks. All cell lines were confirmed as mycoplasm-free. BON-1 and BON-1 R cell lines were cultured in DMEM-F12 supplemented with FCS (10% v/v), L-glutamine (2 mmol/L), fungizone (0.5 mg/L) and penicillin (1 × 10^5^ u/L). The NCI-H727 cell line was cultured in RPMI-1640 supplemented with FBS (10% v/v), L-glutamine (2 mmol/L), penicillin (1 × 10^5^ u/L) and streptomycin (1 × 10^5^ u/L). Cells were incubated in a humidified incubator containing 95% air and 5% CO_2_ with temperature at 37°C.

### Compounds

Everolimus was provided from Novartis Pharma Basel, Switzerland. Lanreotide was provided from Sigma-Aldrich (Darmstadt, Germany). Everolimus and lanreotide powders were dissolved in dimethylsulfoxide (DMSO) at a concentration of 1 × 10^−3^ M and 4.56 × 10^−6^ M, respectively; stock solutions were stored at −20°C and then diluted in DMSO immediately before use. mTOR, p-mTOR^Ser2448^, S6K1, p-S6K1^Thr389^, 4eBP1 and p-4eBP1^Thr70^ antibodies were purchased by Cell Signaling Technology (Beverly, MA, USA); IL-10, IL-6, and TNFα antibodies were supplied from Abcam (Cambridge, UK), while the anti-α-Tubulin antibody from Calbiochem (Jaffrey, NH, USA).

### Patient Inclusion Criteria

According to WHO 2010 classification, 30 patients with intestinal (17 cases), bronchial (10 typical carcinoid), and mammary (3 cases) NETs, under treatment with lanreotide were enrolled. However, cytokine analysis was performed on only 10 patients due to the inadequacy of the sample: 6 patients with intestinal, 2 with bronchial (typical carcinoid) and 2 with breast NETs. The following criteria were required for study selection: histologically confirmed, unresectable, measurable, locally advanced, or metastatic NET either with carcinoid syndrome or functionally inactive; disease progression within 6 months of study entry, based on radiographic images according to the Response Evaluation Criteria in Solid Tumors (RECIST 1.1) (35); expression of somatostatin receptors in the tumor, demonstrated by a positive Octreoscan result; adequate cardiac, hematopoietic, hepatic, and renal function; a wash-out time of at least 4 weeks from any previous treatment with antitumor agents (chemotherapy and/or biological therapy) and 3 months from radiotherapy; no previous treatments with SSAs.

### Treatment Schedule

Slow-release lanreotide (Ipsen S.p.A, Milan, Italy) was administered in a 90-mg deep sc injection every 28 days. No other anticancer medications were allowed during the course of the study.

### Sample Collection

Samples have been collected before treatment with lanreotide started, ten days after the beginning of treatment and then about once a month, for six months, accordingly to clinical practice. The peripheral blood serum of NET patients was centrifuged at 1500 *g* for 10 min; then, it was aliquoted in cryovials and stored at −80°C for the following analyses. All the procedures have been performed with respect to the standard biosecurity and institutional safety measures. Informed consent was obtained from patients to use their samples for research and ethical committee approval was acquired from the Hospital of our University (protocol number 94 of 31st January 2015).

### Cytokine Expression by Cytofluorimetric Analysis

Cytofluorimetric analysis was performed using the BD™ Cytometric Bead Array (CBA) Human Th1/Th2 Cytokine kit II (BD Biosciences, Franklin Lakes, NJ, USA), according to the protocol by supplier. The kit was used to quantitatively measure INF-γ, TNFα, IL-10, IL-6, IL-4, IL-2 in NET serum samples. The analytical properties of BD™ CBA assay was used to evaluate the relevant protein concentrations (pg/mL) in serum: this assay provides a method to capture by flow cytometry a set of analytes with known size beads. Each captured bead is conjugated with a specific antibody. When the capture beads and detector reagent are incubated with an unknown sample containing recognized analytes, sandwich complexes (capture bead + analyte + detection reagent) are formed. These complexes can be measured using flow cytometry detectable in FL3 channel. Six bead populations with distinct fluorescence intensities have been coated with capture antibodies specific for INF-γ, TNFα, IL-10, IL-6, IL-4, IL-2 proteins. The six bead populations were mixed together and resolved in a red channel. The mix of bead was incubated with phycoerythrin (PE)-conjugated antibodies for different cytokines mixture (resolved in FL2 channel). After that, standard curve (0–5000 pg/mL) and the samples were added to bead mix and 1 × 10^4^ events for each sample were acquired. The analysis was performed by BD Accuri™ C6 flow cytometer (BD, Biosciences, Franklin Lakes, NJ, USA), using FCAP Array^TM^ 3.0.1 software. Each sample was processed in triplicate and the data were expressed as mean ± SD.

### MTT Cell Viability Assay

BON-1 and NCI-H727 cells were plated into 96-well plates at a density of 15 × 10^3^ cells/well in 4 replicates. After 24 h, lanreotide was added at increasing concentrations (0.195 to 100 μM). Cells were incubated for 6, 16, and 24 h at 37°C in a humidified atmosphere containing 5% CO_2_. After 6, 16, and 24 h of treatment, cells were used for MTT cell viability assay (Sigma-Aldrich). To evaluate the resistance to everolimus, BON-1 R cells were treated with increasing everolimus concentrations (from 0.62 to 10 μM) and at 24, 48, and 72 h was performed MTT cell viability assay. MTT solution (MTT 5 mg/mL in PBS) was added to the cells and then incubated at 37°C for 1 h. The absorbance of the converted dye was measured at a wavelength of 570 nm, using Victor™ X4 Multilabel Plate Reade (PerkinElmer, MA, USA). Percentage of growth was normalized respect to control cells, represented by untreated cells (100% growth). Each experiment was conducted at least three times and the data were expressed as mean ± SD.

### Western Blot Analysis

Total proteins were homogenized in lysis buffer (Triton 1%, sodium deoxycholate 0.5%, NaCl 0.1 M, EDTA 1 mM, pH 7.5, Na2HPO4 10 mM, pH 7.4, PMSF 10 mM, benzamidine 25 mM, leupeptin 1 mM, aprotinin 0.025 U/mL). Total proteins (50 μg) were separated using Sodium Dodecyl Sulfate—PolyAcrylamide Gel Electrophoresis (SDS-PAGE) at 10% (TGX Stain-Free, BIORAD, Hercules, CA, USA). Proteins were transferred to Nitrocellulose Blotting Membranes 0.2 μm (Trans-Blot Turbo, Mini, BIORAD, Hercules, CA, USA) using the Trans-Blot Turbo® Transfer System (BIORAD, Hercules, CA, USA). Membranes were blocked with 5% milk in T-TBS (0.05% Tween-20, 200 mM Tris-HCl pH 7.5, 1.5M NaCl) for 1 h at room temperature and then incubated overnight in primary antibodies at 4°C. Rabbit monoclonal antibodies for IL-6, TNFα, and mouse monoclonal antibody for IL-10 were used in BON-1 and NCI-H727 cells treated with lanreotide (100 and 200 μM). Rabbit monoclonal antibodies for mTOR, p-mTOR^Ser2448^, S6K1, 4eBP1, p-4eBP1^Thr70^, TNFα, and mouse monoclonal antibody for p-S6K1^Thr389^ and IL-10 were used in BON-1 and BON-1 R cells. The nitrocellulose membranes were washed twice with T-TBS and incubated with secondary antibody in the T-TBS/Milk for 1 h at room temperature. Secondary antibodies include IgG directed against the mouse or rabbit determinants of the first antibody and are conjugated to peroxidase. Membranes were revealed through chemiluminescence reaction reagents (relevant Clarity™ Western ECL Blotting Substrate, BIORAD, Hercules, CA, USA). The quantative analysis was performed with the Image Lab 5.2.1 software (ChemiDoc XRS^+^, BIORAD, Hercules, CA, USA) and the values were normalized on the α-Tubulin expression. Each experiment was conducted three times and the data were expressed as mean ± SD.

### Statistical Analyses

Statistical analysis was performed using Graphpad 5 software (Graphpad Software, La Jolla, CA, USA) and the results were considered statistically significant at a level of *P* ≤ 0.05. IC_50_ concentrations were calculated by Spline method. Differences between treatment and control cells were analyzed using a one-way ANOVA followed by a multiple comparative test (Newman-Keuls).

## Results

### Patient Characteristics and Serum Cytokine Determination

We performed a cytofluorimetric analysis aimed to evaluate the parallel expression of IFN-γ, TNFα, IL-10, IL-6, IL-4, IL-2 in the serum of 10 patients (8 males and 2 females, mean age: 67.4 ± 3.7, median age: 68) with intestinal (6 patients), bronchial (typical carcinoid, 2 patients), and breast NETs (2 patients) receiving treatment with lanreotide. The patients enrolled in the study were at least for 1 month without any other specific cancer treatments and all the values were compared to the baseline in absence of lanreotide administration. During the study, 1 complete response (CR) lasting 5 months was recorded in a patient with intestinal NET, 2 partial responses (PRs) lasting 12 and 13 months, respectively, in 2 patients with intestinal NETs and 2 stable diseases (SDs) lasting 6 and 5 months in other 2 patients with intestinal NETs, respectively (Table 1). A progression disease (PD) was recorded in a patient with intestinal NET, in the 2 patients with bronchial NETs and in the 2 patients with mammary NETs (Table 1). All the patients had liver metastases at the beginning of the treatment and only 3 patients are still presently alive: all with intestinal NETs with an overall survival (OS) of 47, 53, and 50 months, respectively (Table 1). Only the 2 patients with mammary NETs received previous chemotherapy lines while all the remaining patients did not receive any previous medical and/or radiation treatments.

**Table 1:** Clinical characteristics of patients with NETs.

**Patient**	**Age**	**Gender**	**Tumor localization**	**Metastatic sites**	**Previous medical treatments**	**OS (months)**	**PFS (months)**	**Response to treatment**
1	60	M	Intestinal NET	Liver, nodes	None	30	6	SD
2	62	M	Intestinal NET	Liver	None	47+	5	CR
3	71	M	Intestinal NET	Liver, Mesenteric	None	53+	12	PR
4	67	M	Intestinal NET	Liver, nodes	None	50+	13	PR
5	68	M	Intestinal NET	Nodes, liver, heart	None	38	2	PD
6	67	M	Intestinal NET	Nodes, liver	None	40	5	SD
7	71	M	Bronchial NET	Liver, bone, nodes	None	96	7	PD
8	70	M	Bronchial NET	Liver, bone	None	90	6	PD
9	70	F	Mammary NET	Liver, bone	3 Chemotherapy lines	9	3	PD
10	68	F	Mammary NET	Liver, bone	2 Chemotherapy lines	8	2	PD

*OS, overall survival; PFS, progression-free survival; SD, stable disease, CR, complete response; PR, partial response; PD, progression disease*.

**Figure 1:**
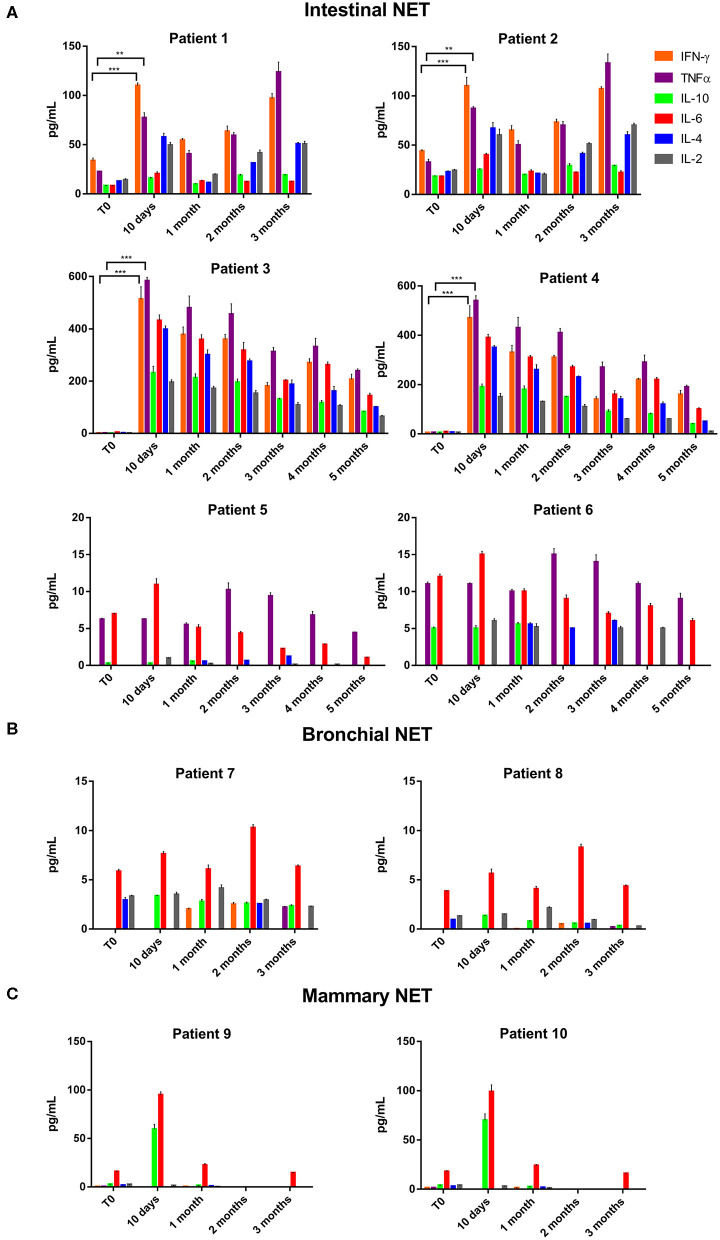
INF-γ, TNFα, IL-10, IL-6, IL-4, and IL-2 expression in serum of patients with: **(A)**, intestinal NET (patient 1-6); **(B)**, bronchial (patients 7 and 8) and **(C)**, mammary NETs (patients 9 and 10) after treatment with lanreotide by cytofluorimetric analysis. Each experiment was repeated three times and the data are representative samples of the total number of patients analyzed and shown as mean ± SD. ** *P* ≤ 0.01; *** *P* ≤ 0.001.

The data of the analyzed patients show a different trend of serum cytokine concentrations in relation to the primary tumor site. In details, in all the analyzed patients and independently from the tumor site of origin, the levels of IL-6 increased after 10 days of treatment with lanreotide and decreased in the following six months; whereas the levels of IL-2 and IL-10 raised only in some patients. On the contrary, the levels of IL-4 decreased already at 10 days, as compared to baseline untreated control in sera from the patients affected by bronchial NETs. Only in intestinal NETs, IFN-γ and TNFα increased after 10 days of treatment with lanreotide (Figure 1A). Therefore, our data suggest that intestinal NETs are characterized by a higher early and more significant Th1 than Th2 response that could be associated to a greater sensitivity to the immune-mediated effects of lanreotide. Interestingly, all the clinical responses were recorded in intestinal NETs with 3 patients still alive after about 50 months from the beginning of the treatment.

On the other hand, IFN-γ and TNFα were not detectable in sera from patients with bronchial and mammary NETs (Figures 1B,C). The latter data suggest that bronchial and mammary NETs have an inflammatory and immunological micro-environment poorly responsive to lanreotide. Interestingly, all these patients experienced a PD with a limited OS in heavily pre-treated mammary NETs.

### Lanreotide Effect on Cell Viability in Bronchial and Pancreatic NET Cell Lines

**Figure 2:**
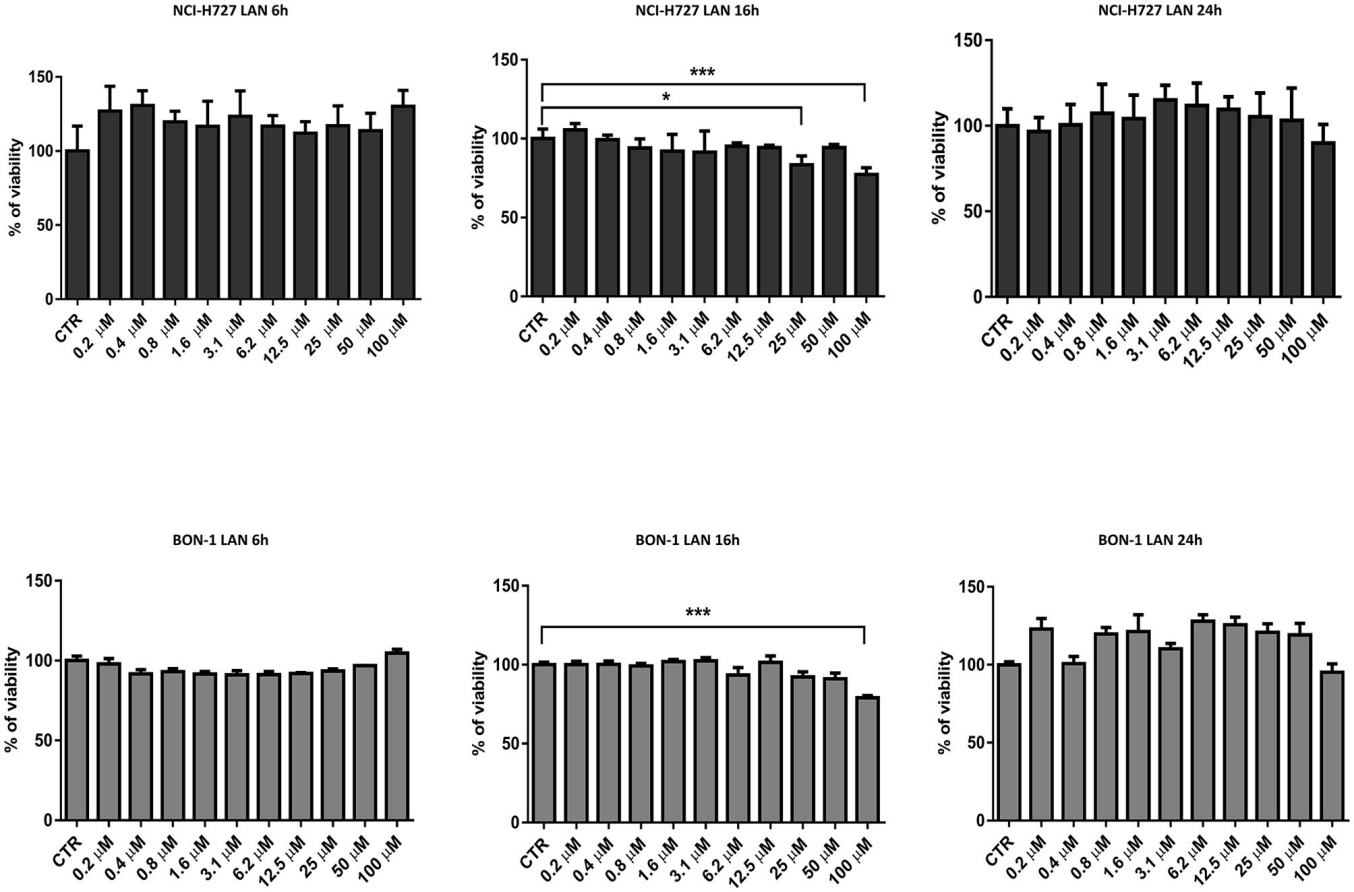
Cell viability evaluated by MTT, after 6, 16 and 24 h of treatment with lanreotide (from 0.195 to 100 μM) in NCI-H727 and BON-1 cell lines. Each experiment was repeated three times and shown as mean ± SD. * *P* ≤ 0.05; *** *P* ≤ 0.001.

Thereafter, we evaluated the *in vitro* effects of lanreotide on NCI-H727 bronchial and BON-1 pancreatic NET cell lines expressing SSRs. The viability of these cells exposed to escalating lanreotide concentrations [range 0.195–100 μM] was evaluated after 6, 16, and 24 h from the beginning of the exposure to lanreotide with MTT cell viability assay. In both cell lines, lanreotide caused minimal cytostatic effects at 16 h. In particular, NCI-H727 cell line showed a reduction in viability of 17% (*P* ≤ 0.05) and 23% (*P* ≤ 0.001) when exposed to a lanreotide concentration of 25 and 100 μM, respectively; while BON-1 cell line showed no effect up to 25 μM and 21% (*P* ≤ 0.001) reduction in viability at 100 μM (Figure 2). The exposure of the two cell lines to lanreotide for more prolonged times (up to 6 days) determined a lost of the growth inhibitory effects (data not shown).

### Lanreotide Effects on Inflammatory Cytokine Expression by Bronchial and Pancreatic NET Cell Lines *in vitro*

Considering the difficulty to evaluate the effects of treatment with lanreotide on the cytokine levels in rare tumors, such as NETs, we analyzed the protein expression by Western Blotting on stable and reproducible NET models, NCI-H727 and BON-1 cell lines, exposed for 24 h to lanreotide at the final concentration of 100 and 200 μM, respectively. The cytostatic effect induced by treatment with lanreotide on the cells was very minimal thus, to evaluate the protein expression of cytokines, we increased the concentration to 200 μM.

**Figure 3:**
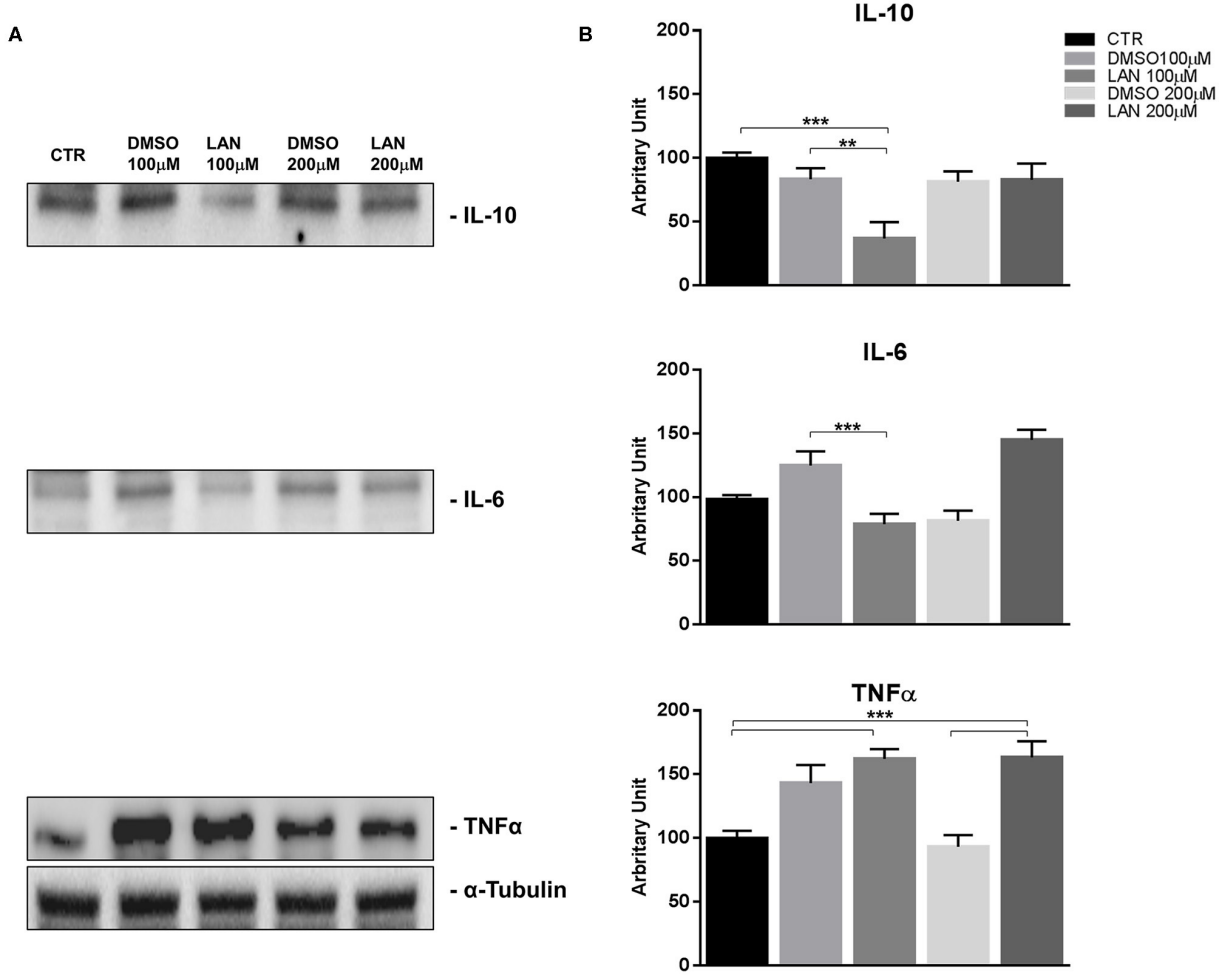
IL-10, IL-6, TNFα expression in BON-1 cell line after treatment with 100 and 200 μM lanreotide. **(A)**, Western blotting analysis of IL-10, IL-6, TNFα. **(B)**, Quantitative analysis reported as arbitrary units showing the variation of IL-10, IL-6, TNFα expression levels compared to a-Tubulin, as housekeeping protein, using Image Lab 5.2.1 ChemiDoc XRS+ (BIORAD). The results are shown as mean ± SD of three independent experiments. ** *P* ≤ 0.01; *** *P* ≤ 0.001.

**Figure 4:**
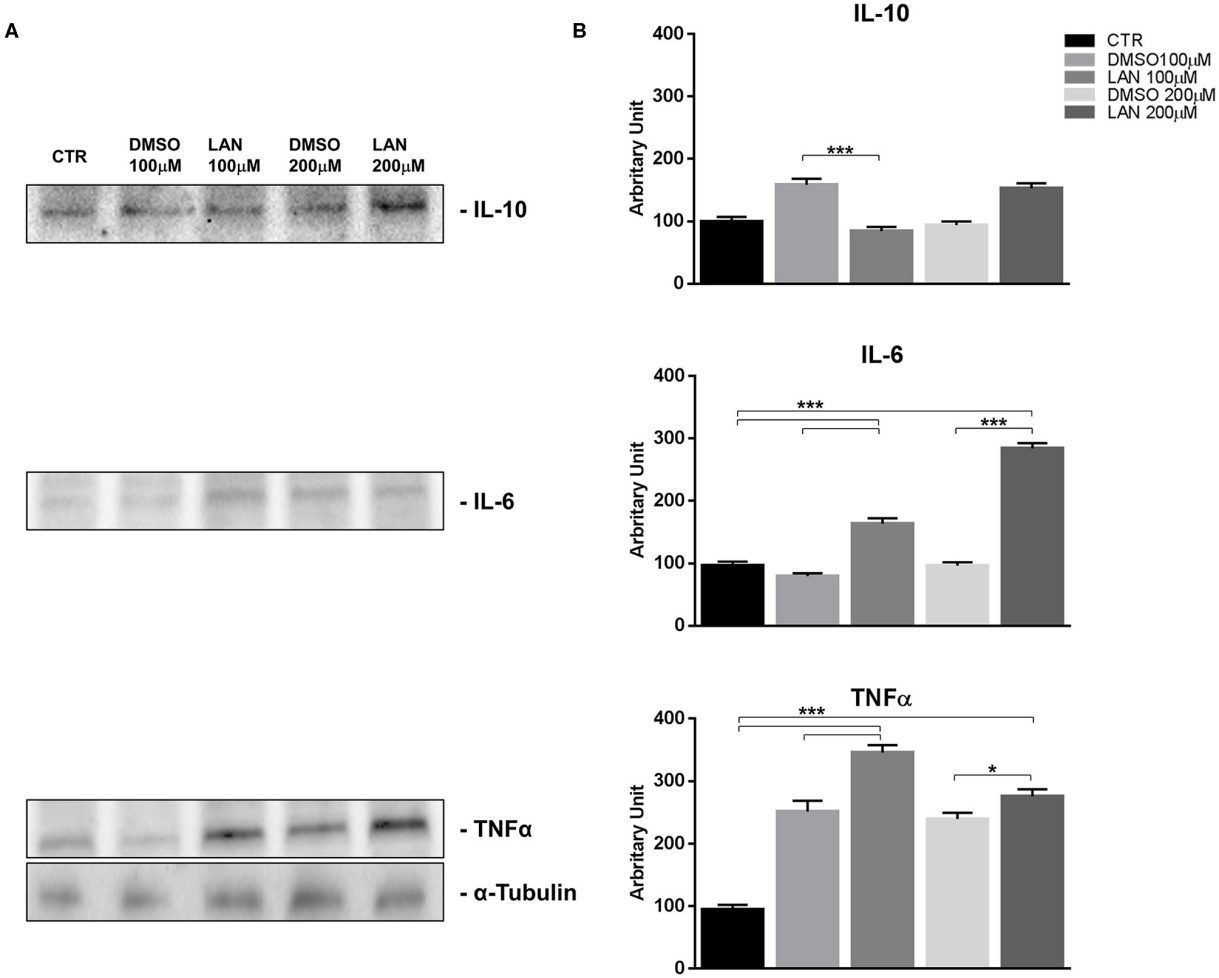
IL-10, IL-6, TNFα expression in NCI-H727 cell line after treatment with 100 and 200 μM lanreotide. **(A)**, Western blotting analysis of IL-10, IL-6, TNFα. **(B)**, Quantitative analysis reported as arbitrary units showing the variation of IL-10, IL-6, TNFα expression levels compared to α-Tubulin, as housekeeping protein, using Image Lab 5.2.1 ChemiDoc XRS+ (BIORAD). The results are shown as mean ± SD of three independent experiments. * *P* ≤ 0.05; *** *P* ≤ 0.001.

Our experiments in BON-1 cells derived from a pancreatic NET revealed significant treatment-related increase in TNFα synthesis paralleled by a significant reduction in IL-6 and IL-10 expression; this cytokine profile reflects the ability of lanreotide to induce a Th1 cytotoxic immune-response (Figures 3A,B). On the other hand, exposure of NCI-H727 bronchial NET cell line to lanreotide induced a treatment-related increase of IL-6 and of TNFα expression paralleled by a decrease in the expression of IL-10 (Figures 4A,B). The above-mentioned findings were not recorded in tumor cell lines, untreated or exposed to DMSO (100 and 200 μM) used as drug vehicle negative control. Therefore, the effects obtained *in vitro* on the two cell lines were on line with those recorded *ex vivo* on patient sera.

### Lanreotide Effects on a Pancreatic NET Cell Line Resistant to Everolimus

**Figure 5:**
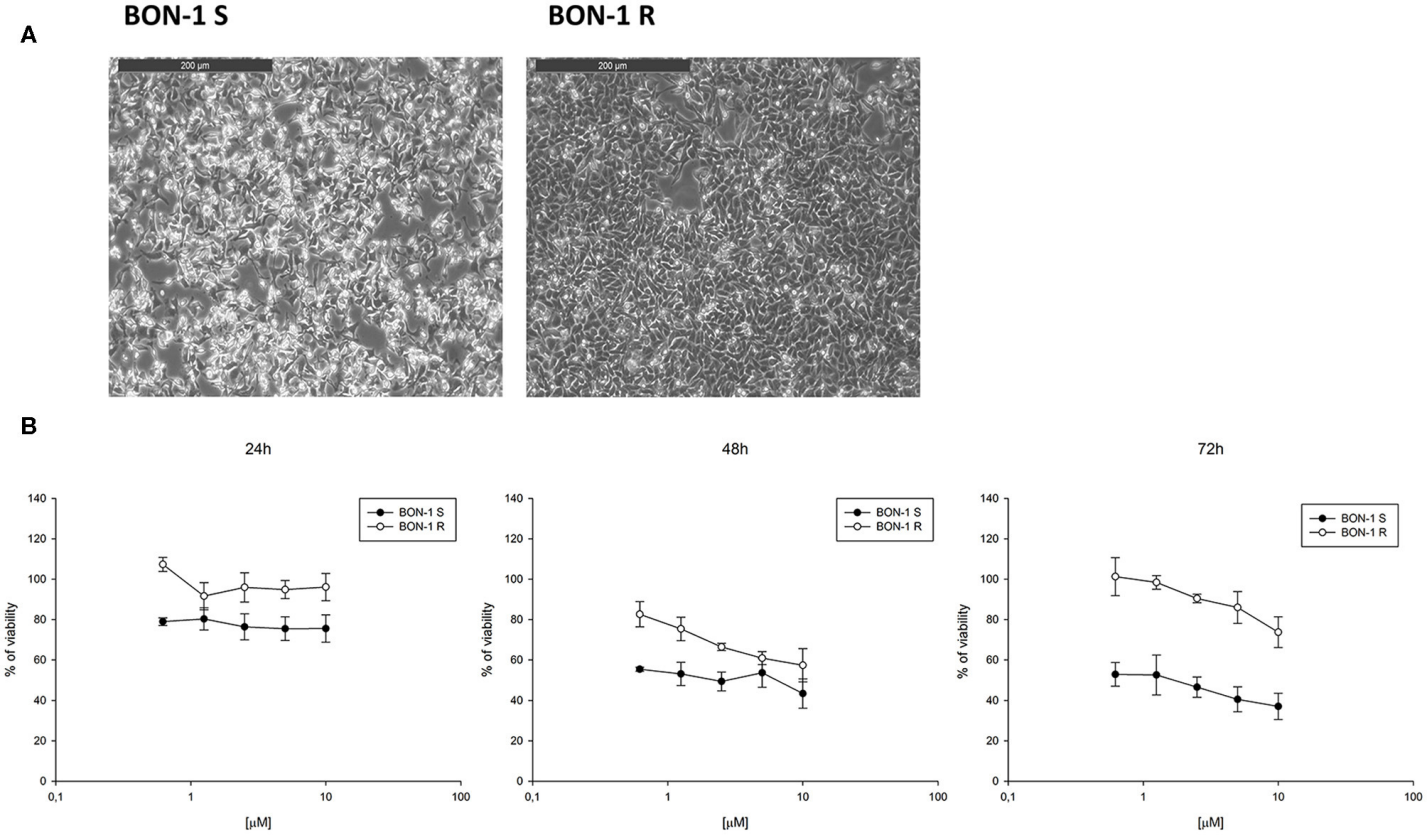
**(A)**, Morphological analysis of BON-1 cell line, sensitive (S) and resistant (R) to everolimus. **(B)**, Cell viability by MTT assay on sensitive and resistant BON-1 cell line after 24, 48, and 72 h of treatment with everolimus (from 0.625 to 10 μM). Each experiment was repeated three times and shown as a mean ± SD.

**Figure 6:**
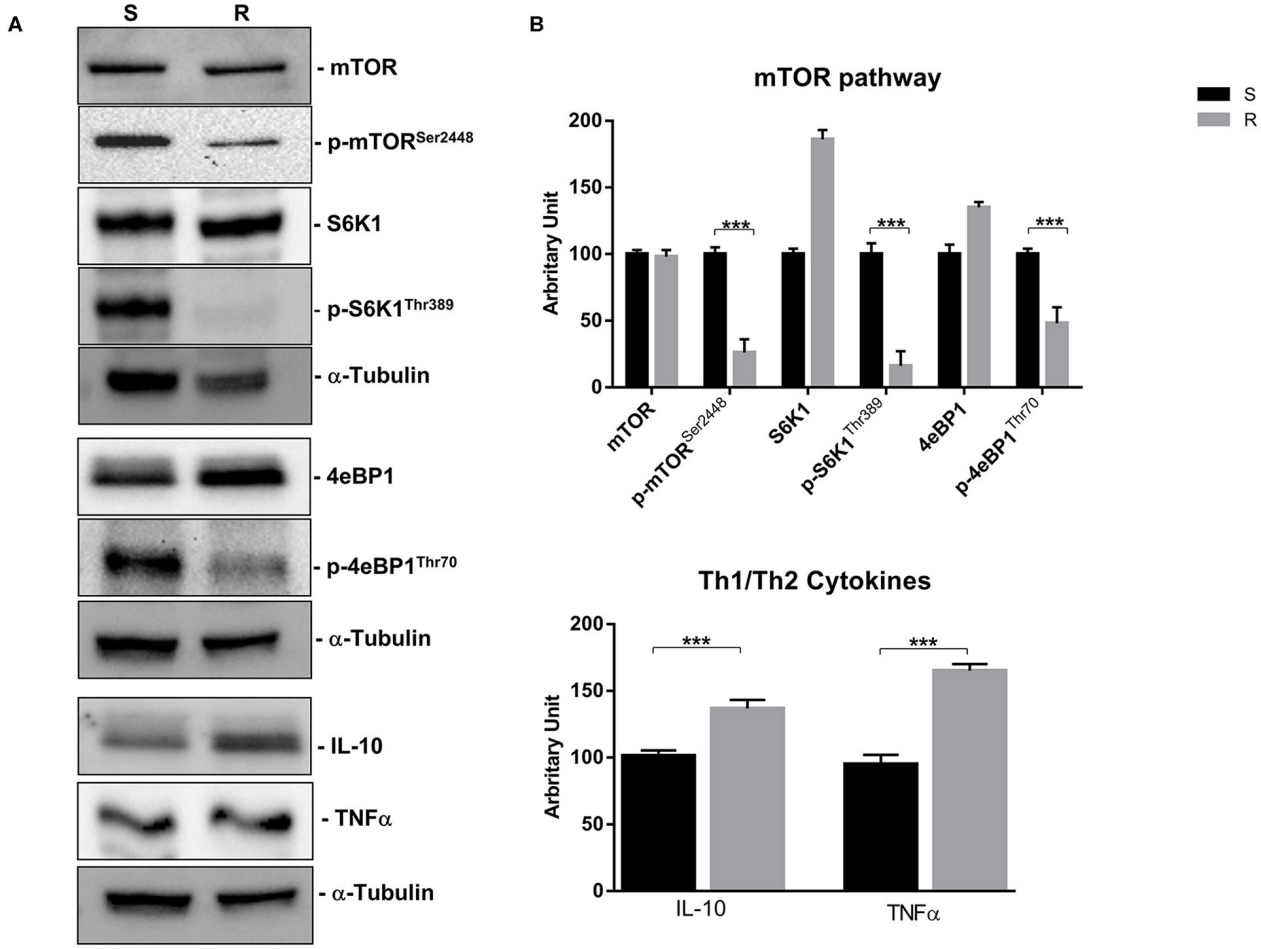
mTOR pathway (mTOR, p-mTOR^Ser2448^, S6K1, p-S6K1^Thr389^, 4eBP1, and p-4eBP1^Thr70^) and Th1 cytokines (IL-10 and TNFα) proteins expression in sensitive and resistant BON-1 cell line. **(A)**, Western blotting analysis of mTOR, p-mTOR^Ser2448^, S6K1, p-S6K1^Thr389^, 4eBP1, p-4eBP1^Thr70^, IL-10, and TNFα. **(B)**, Densitometric analysis of bands showing the variation of the expression levels of the mTOR pathway and Th1/Th2 cytokines compared to α-Tubulin, as housekeeping protein, using Image Lab 5.2.1 ChemiDoc XRS+ (BIORAD). The results are obtained from three independent experiments and plotted as mean ± SD. *** *P* ≤ 0.001.

In order to evaluate the ability of lanreotide to overcome tumor cell resistance to mTOR inhibitors, we generated an everolimus-resistant BON-1 pancreatic NET cell line. At this purpose, we performed a long lasting (8 weeks) culture of these cells with escalating everolimus concentrations (range 1.25–10 μM). The resistance to everolimus was demonstrated by performing MTT cell viability assay after 24, 48, and 72 h of exposure to everolimus (concentration range: 0.62–10 μM) (Figure 5B). Additional experiments were also performed to demonstrate mTOR pathway inactivation in the resistant BON-1 cell line (R) compared to sensitive cells (S) with no other detectable morphological and phenotypic changes (Figures 5A, 6A,B).

Indeed, a significant reduction (from 5 to 2-fold) of mTOR phosphorylation was detected as well as of the activity of its downstream effectors (S6K1 and 4eBP1). Noteworthy, the reduction of molecular target activity is one of the best-known mechanisms of resistance to target-based agents.

**Figure 7:**
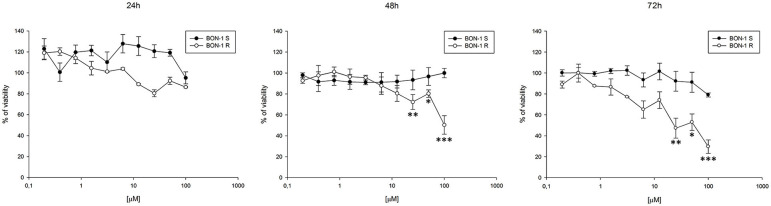
Analysis of cell viability by MTT assay on sensitive (S) and resistant (R) BON-1 cell line after 24, 48, and 72 h of treatment with lanreotide (from 0.195 to 100 μM). Each experiment was repeated three times and shown as mean ± SD. * *P* ≤ 0.05; ** *P* ≤ 0.01; *** *P* ≤ 0.001.

Thereafter, we evaluated the antitumor effects of lanreotide on cells resistant to everolimus compared with the sensitive BON-1 parental cells in 24, 48, and 72 h MTT cell viability assays. In these experiments, we found a significant dose-time dependent anti-tumor effect of lanreotide only on the tumor cells resistant to everolimus (Figure 7). This antitumor effect of lanreotide was maximal after 72 h of exposure with a 50% proliferative inhibition (IC_50_) of 25 μM. These data suggest that lanreotide blocks transduction pathways alternative to mTOR in everolimus-resistant tumor cells and sensitizes these cells to the antiproliferative effects induced by chronic exposure to mTOR inhibitors. We have also evaluated the effects of everolimus-induced resistance on the expression of membrane-associated isoform of TNFα and of IL-10 in these cells. We have found an increase of both cytokines in everolimus-resistant cells as compared with sensitive ones (Figures 6A,B). The previous data suggest that the resistant phenotype confers an increase of TNFα, a Th1 cytokine making the cells likely more sensitive to the inhibitory activity of lanreotide.

## Discussion

Neuroendocrine tumors (NETs) are neoplasms that arise from cells of the so-called “Neuroendocrine Diffuse System” with morphological and functional features similar to neurons, although they are without axons or synapses.

The most relevant therapeutic strategies for this disease consist in the use of molecules able to interfere with the main pathways involved in the pathogenesis and function of NET cells. These approaches regard the use of SSAs, including octreotide and lanreotide as well as mTOR inhibitors like everolimus.

Several studies have shown that neuroendocrine tumor cells may synthesize cytokines (CKs) (29) that in turn, act as tumor growth factors (i.e., IL-8) and also affect the immunosurveillance efficacy (30). Th1 immunity is gaining an important role in cancer therapy and weak Th1 responses are suggestive of poor treatment response and prognosis (36). This role for Th1 immunity is likely due to the CD4+ T helper cell function in stimulating both innate and adaptive components of immune system in response to tumors through direct cytotoxic tumoricidal activity, modification of antitumor cytokine responses and potentiation of long term immunologic memory (37). In line with other scientific findings, we have recorded that lanreotide treatment is able to restore a Th1 phenotype in patients with intestinal NETs as showed by a significant increase in TNFα and IFN-γ. Additionally, we also showed that all patients continue to produce high levels of Th2 cytokines probably as consequence of a higher representation of lanreotide-resistant cells and previous different treatments. In this light, both IFN-γ and TNFα were not detectable in patients with mammary and bronchial NETs suggesting that other cytokines such as IL-17A as well as IL-8 might be involved in the activation pathways of these tumors or that simply their expression may not be modulated by lanreotide for a different expression of sensitive SSRs. The modulation of IFN-γ production observed in patients with NETs was not tested *in vitro* experiment due to the inability of tumor cells to produce this cytokine. In line with these results, we also found that the exposure to lanreotide is able to upregulate the production of TNFα and downregulate the expression of IL-6 and IL-10 only in the pancreatic-derived cells, while lung NETs showed an opposite profile. On this light, we have previously demonstrated that lanreotide can increase the activity of IL-2-activated peripheral blood mononuclear cells against a cellular model of NET of the thyroid, the medullary thyroid carcinoma (MTC) TT cell line. Moreover, we have showed that a combination schedule based upon the concomitant administration of recombinant IL-2 and lanreotide in a series of 6 patients affected by metastatic MTC was an active and safe treatment (38). Another previous therapeutic approach in this cancer subset was the combination between lanreotide and interferon α (IFNα) that gave some clinical results even if less promising (39). The previous and present data encourage the exploration of new strategies in NET based upon the combined use of immunological checkpoint inhibitors and lanreotide. In this context, everolimus has gained an important role in the treatment guidelines of NET, but one-third of NET patients invariably show primary resistance to everolimus, while the majority of the patients develop acquired resistance and disease progression within 24 months (25). In our study, we have investigated the effects of lanreotide in pancreatic cell lines made resistant to everolimus *in vitro*. We have characterized this cell line for the activity and expression of the mTOR-dependent pathway and we have found, as expected, an increased activity of the targets as an explanation of the resistance. In this experimental model, our data suggest that the parental pancreatic NET cells were highly resistant to the antiproliferative effects induced by lanreotide compared to everolimus resistant derivative cells. The use of lanreotide in this setting could have a new indirect mechanism of action which could overcome the resistance to everolimus. We have also demonstrated an increased expression in everolimus-resistant cell line, as compared to the parental counterpart of TNFα suggesting that a Th1 response could be useful in immunological integrated treatment strategies. IL-10 showed the same expression profile in NET cell line resistant to everolimus compared to sensitive one. In fact, as reported by Stassi et al. the cancer resistance to chemotherapeutic drugs is related to the autocrine production of IL-4 and IL-10 (40).

Experiments are currently in progress to evaluate the cytokine expression modulation in the resistant cells by lanreotide as well as its effects on the expression of the different SSTR subtypes.

Overall our results suggest that lanreotide treatment of intestinal NET tumors promotes the occurrence of a Th1 cytotoxic phenotype, a fact that may represent a solid rationale to combine lanreotide with immune-oncological strategies which include immunodulating cytokines (IL-2, IFNα) or PD-1/PD-L1 inhibitors. Additionally, our *in vitro* results suggest that lanreotide may be considered as an efficient rescue treatment when everolimus resistance occurs in patients with intestinal NETs.

## Data Availability Statement

The datasets generated for this study are available on request to the corresponding author.

## Ethics Statement

Written informed consent was obtained from the individuals for the publication of any potentially identifiable images or data included in this article.

## Author Contributions

CS and AL have contributed equally to this work, prepared the manuscript, and assembled figures. FR, CM, MB, GM, ACo, GV, and PC conceived and designed the experiments. AG performed experiments and analyzed data. RM clinically evaluated patients enrolled for the study. AMC conducted the experiments. AF, AN, JF, MG, MC, and ACa revised the manuscript. All authors contributed to the article and approved the submitted version.

## Conflict of Interest

The authors declare that the research was conducted in the absence of any commercial or financial relationships that could be construed as a potential conflict of interest. The reviewer PG declared a past co-authorship with several of the authors AL, AMC, and MC to the handling editor.
